# The Combined Prognostic Value of ^18^ F‐FDG PET/CT Metabolic Parameters of Immune Organs and Hematological Immune‐Related Markers in Patients With Locally Advanced Cervical Cancer

**DOI:** 10.1002/cam4.70650

**Published:** 2025-02-06

**Authors:** Yi Li, Xin Wang, Yuanlin Li, Wanhu Li, Defeng Liu, Longxiang Guo, Xiuli Liu, Zhichao Li, Ao Liu, Minghuan Li

**Affiliations:** ^1^ Department of Radiation Oncology, Shandong Cancer Hospital and Institute Shandong First Medical University and Shandong Academy of Medical Sciences Jinan China; ^2^ School of Clinical Medicine Shandong Second Medical University Weifang China; ^3^ Department of PET/CT Center, Shandong Cancer Hospital and Institute Shandong First Medical University and Shandong Academy of Medical Sciences Jinan Shandong China; ^4^ Department of Radiation Oncology, Shandong Cancer Hospital, Cheeloo College of Medicine Shandong University Jinan China; ^5^ Department of Oncology Dongying People's Hospital Dongying Shandong China; ^6^ Department of Radiation Oncology, Qilu Hospital, Cheeloo College of Medicine Shandong University Jinan China

**Keywords:** ^18^F‐FDG PET/CT, cervical cancer, prognosis, standardized uptake value, systemic immunity

## Abstract

**Background:**

This study aimed to explore the prognostic value of fluorine‐18 fluorodeoxyglucose positron emission tomography/computed tomography (^18^F‐FDG PET/CT) metabolic parameters of immune organs and hematological immune‐related markers for patients with locally advanced cervical cancer (LACC) undergoing concurrent chemoradiotherapy (CCRT), and to establish prognostic nomograms based on these potential biomarkers.

**Methods:**

A total of 180 patients with LACC undergoing CCRT were retrospectively reviewed and randomly divided into training and validation groups at a 7:3 ratio. Cox regression analysis was performed to identify independent prognostic factors for progression‐free survival (PFS) and overall survival (OS) from hematological immune‐related markers and ^18^F‐FDG PET/CT metabolic parameters of the primary tumor, spleen, and bone marrow (BM). Nomograms were developed and evaluated using receiver operating characteristic curves, concordance index (C‐index), calibration curves, and decision curve analysis (DCA). Spearman correlation analysis was used to assess the relationships among metabolic parameters.

**Results:**

Multivariable analysis identified International Federation of Gynecology and Obstetrics (FIGO) stage, neutrophil‐to‐lymphocyte ratio (NLR), and spleen maximum standardized uptake value (SUV_spleen_) as independent prognostic factors for PFS. For OS, the independent prognostic factors were FIGO stage, NLR, metabolic tumor volume, and SUV_spleen_. The nomograms demonstrated better prognostic performance for PFS (area under curve [AUC]: 0.875 and 0.862; C‐index: 0.809 and 0.775) and OS (AUC: 0.858 and 0.814; C‐index: 0.828 and 0.792) in the training and validation groups. Calibration curves and DCA indicated that the nomograms have good predictive accuracy and clinical utility. Spearman correlation analysis revealed significant positive correlations among total lesion glycolysis, SUV_spleen_, SUV_BM_, and platelet‐to‐lymphocyte ratio.

**Conclusion:**

The nomograms based on metabolic parameters of immune organs and hematological immune‐related markers demonstrated high predictive value for patients with LACC undergoing CCRT. The observed correlations between the metabolic parameters of the primary tumor and immune organs suggest a widespread disturbance of systemic immunity caused by the tumor.

## 1 Introduction

Cervical cancer is the most common malignancy of the female reproductive system, with over half of newly diagnosed cases classified as locally advanced cervical cancer (LACC) [1, 2]. The standard treatment for LACC is definitive concurrent chemoradiotherapy (CCRT) [3, 4]. However, approximately 40% of patients experience recurrence after CCRT, affecting survival outcomes [5, 6, 7]. Currently, the primary method for assessing the risk of recurrence and guiding clinical treatment is the International Federation of Gynecology and Obstetrics (FIGO) staging system for cervical cancer [8, 9, 10]. However, patients with the same FIGO stage may experience different clinical outcomes due to the biological heterogeneity of the tumor. Therefore, developing a more effective prognostic stratification model is necessary.

Systemic immunity significantly impacts tumor treatment response and clinical outcomes [11, 12]. After radiotherapy, effective tumor control relies not only on the direct cytotoxic effect of radiation on tumor cells but also on radiation‐induced immune responses in the tumor and the systemic immune status of patients. Liang et al. [13] demonstrated that progression‐free survival (PFS) after radiotherapy is influenced by radiation‐induced tumor equilibrium (RITE), which is characterized by the simultaneous presence of active tumor cell proliferation and significant immune infiltration. It is essential to conduct a comprehensive quantitative analysis of the tumor's biological characteristics and the systemic immune status to accurately identify patients with LACC who are at high risk of recurrence.

As a functional imaging modality, Fluorine‐18 fluorodeoxyglucose positron emission tomography/computed tomography (^18^F‐FDG PET/CT) has been extensively used for diagnosing, staging, and prognostic assessment for cervical cancer [14]. Increasing research has indicated that the metabolic parameters of primary tumors, spleen, and bone marrow (BM) are associated with tumor outcomes in cervical cancer [15, 16, 17, 18]. Kidd et al. [18] found that a higher maximal standardized uptake value (SUV) of the primary cervical tumor was associated with poorer survival. De Jaeghere et al. [15] found that an increased spleen‐to‐liver uptake ratio (SLR) correlated with worse disease‐free survival. Notably, patients with a higher SLR exhibited greater infiltration of CD20+ and CD68+ immune cells in tumor tissue. Similarly, Lee et al. [16] observed that a higher bone marrow‐to‐liver uptake ratio (BLR) was linked to worse distant recurrence‐free survival. Additionally, they found a positive correlation between SUV_BM_ and BLR, with neutrophil‐to‐lymphocyte ratio (NLR). These studies highlighted the prognostic value of metabolic parameters in these immune organs and their connection to systemic immunity. However, tumor progression often involves widespread changes in systemic immunity, with a dynamic network of immune communication among the spleen, bone marrow, and blood [19, 20]. Therefore, to provide a more comprehensive assessment of systemic immunity and enhance the accuracy of prognosis prediction, we evaluated the metabolic parameters of these immune‐related organs collectively.

Additionally, increased FDG uptake in immune‐related organs indicates activation and responsiveness to systemic inflammatory reactions associated with antitumor immunity during tumor progression [16, 21, 22]. The status of systemic inflammation can be reflected by hematological markers, which have been shown to significantly correlate with survival outcomes [23, 24, 25]. This present study aimed to explore the prognostic value of hematological immune‐related markers and metabolic parameters of both the primary cervical tumor and immune organs, including spleen and bone marrow, to predict PFS and overall survival (OS) in patients with LACC who underwent CCRT. Additionally, the study sought to establish prognostic nomograms based on these potential biomarkers. In this way, tumor heterogeneity and systemic immune status can be comprehensively assessed, thereby guiding clinicians to make more accurate clinical decisions and improving patient prognosis in the era of immunotherapy.

## 2 Materials and Methods

### 2.1 Patients

This study retrospectively included 180 patients who were newly diagnosed with LACC and underwent a complete course of CCRT between December 2015 and December 2022 at Shandong Cancer Hospital and Institute. The inclusion criteria included: (1) age ≤ 75 years; (2) FIGO stage IB3‐IVA (2018 edition); and (3) availability of pre‐treatment ^18^F‐FDG PET/CT imaging and comprehensive clinical and pathological data. The exclusion criteria included: (1) presence of other tumors; (2) acute infection or autoimmune disease; and (3) loss to follow‐up or failure to adhere to review requirements. The study was approved by the Institutional Review Board (IRB) of Shandong Cancer Hospital.

### 2.2 Baseline and Hematological Parameters

The collected baseline data included age, FIGO stage, maximum tumor diameter, pathology, involvement of pelvic and para‐aortic lymph nodes, and radiation dose. Hematological parameters were collected within 1 week pre‐treatment to obtain immune‐related markers [25, 26, 27]. The calculation formulas for these markers are as follows:

$$\text{neutrophil}-\text{to}-\text{lymphocyte ratio}\;\left(\mathrm{NLR}\right)=\frac{\text{neutrophil count}}{\text{lymphocyte count}}$$

$$\text{platelet}-\text{to}-\text{lymphocyte ratio}\;\left(\mathrm{PLR}\right)=\frac{\text{platelet count}}{\text{lymphocyte count}}$$

$$\text{lymphocyte}-\text{to}-\text{monocyte ratio}\;\left(\mathrm{LMR}\right)=\frac{\text{lymphocyte count}}{\text{monocyte count}}$$

$$\begin{array}{c} \text{systemic immune}-\text{inflammation index}\;\left(\mathrm{SII}\right)=\frac{\text{platelet count}*\text{neutrophil count}}{\text{lymphocyte count}};\\ \text{systemic inflammation response index}\;\left(\text{SIRI}\right)=\frac{\text{monocyte count}*\text{neutrophil count}}{\text{lymphocyte count}} \end{array}$$

### 2.3 Treatment and Follow‐Up

All patients underwent CCRT according to domestic and international guidelines and standards [3, 28]. The radiotherapy regimen included external beam radiotherapy (EBRT) and brachytherapy. EBRT was administered in a dose of 45–50.4 Gy, delivered in fractions of 1.8–2.0 Gy, with an additional 10–15 Gy for positive pelvic lymph nodes. Brachytherapy was delivered at a dose of 30 to 40 Gy in 6–8 fractions. The cumulative dose of EBRT and brachytherapy reached 80–90 Gy. The chemotherapy regimen primarily involved weekly cisplatin (40 mg/m^2^). Follow‐up assessments were conducted as follows: during the first 2 years after CCRT, patients were evaluated every 3 months; from the third to fifth years, assessments occurred every 6 months; thereafter, evaluations were conducted annually. In this study, PFS was calculated from the end of CCRT to the first instance of disease recurrence, metastasis, death, or until the latest follow‐up. OS was defined as the time until death from any cause.

### 2.4 ^18^F‐FDG PET/CT Examination

Baseline ^18^F‐FDG PET/CT examination was performed within 2 weeks before CCRT. Patients fasted for at least 6 h prior to the scan to ensure normal glucose levels. They received an intravenous injection of ^18^F‐FDG at 4.4 MBq/kg and then rested for about 60 min before imaging. Scans were conducted using an integrated inline PET/CT system (Gemini TF Big Bore, Philips Healthcare). Whole‐body CT scans were done first (dose modulation: 150 mAs, 130 kV, matrix size 512 × 512, slice thickness 3 mm), followed by a 1‐min PET scan per bed (matrix size: 144 × 144). The PET images were reconstructed using iterative ordered subset expectation maximization methods. Subsequently, these images were integrated with CT images to produce whole‐body coronal, sagittal, and transverse views.

### 2.5 ^18^F‐FDG PET/CT Image Analysis

Without prior knowledge of the patients' pathological backgrounds or clinical outcomes, all volume of interest (VOIs)—including the primary cervical tumor, spleen, liver, and bone marrow—were delineated in the pre‐treatment ^18^F‐FDG PET/CT examination by two experienced radiation oncologists. These delineations were then reviewed and verified, slice by slice, by an experienced nuclear medicine physician. At first, the VOIs for the primary cervical tumor were delineated, and the maximum standardized uptake value of cervical cancer (SUV_cervix_), total lesion glycolysis (TLG), and metabolic tumor volume (MTV) were measured [29]. The MTV was determined using a margin threshold of 40% of the maximum standardized uptake value [15, 30, 31]. Furthermore, spheroid VOIs with diameters of 5 and 3 cm were positioned at the center of the right lobe of the liver and the spleen, respectively [15, 21]. Using these VOIs, we measured the maximum standardized uptake values for the liver (SUV_liver_) and spleen (SUV_spleen_) in each patient. Subsequently, the spleen‐to‐liver ratio (SLR) was calculated by dividing SUV_spleen_ by SUV_liver_.

Finally, VOIs were delineated on each vertebral body from the L3‐5 and S1‐2 spines. Given the good reproducibility of the 75% SUVmax threshold among subjects with representative lesions, automatic isocontours set at 75% of SUVmax were used to measure the SUVmean for each VOI [32]. The SUVmean of the selected vertebral body was calculated and defined as SUV_BM_. Finally, the BM‐to‐liver ratio (BLR) was calculated by dividing SUV_BM_ by SUV_liver_ [16].

### 2.6 Statistical Analysis

Age and EBRT dose were dichotomized using median values as cutoff values. Other continuous variables were dichotomized based on the optimal cutoff value determined from receiver operating characteristic (ROC) curves for PFS. Patients were randomly divided into training and validation groups in a ratio of 7:3. The *t*‐test (for continuous variables) and the chi‐squared test (for categorical variables) were used to evaluate differences in baseline characteristics between the groups, with Fisher's exact test applied to assess *p*‐values where appropriate. Univariable and multivariable Cox regression analysis were used to identify independent prognostic factors for PFS and OS. Kaplan–Meier (KM) analysis was used to compare survival rates and plot survival curves, while the Log‐rank test was performed to assess the significance of differences between the survival curves.

Nomograms for PFS and OS were developed based on the results of the Cox regression analysis using the ‘rms’ package in R software [33]. ROC curves and concordance indices (c‐index) were utilized to evaluate the nomograms and compare them with FIGO stage, which is the primary method used to guide treatment for LACC [8, 9, 10]. The prognostic accuracy and clinical utility of the nomograms were evaluated using calibration curves and decision curve analysis (DCA). Risk stratification was performed based on the optimal cutoff value of the nomograms scores determined from the ROC curves. The relationships between different variables were assessed using Spearman correlation analysis. All statistical analyses were performed using SPSS 27.0.1 and R software 4.3.2. All statistical tests were two‐sided, and a *p*‐value of < 0.05 was considered statistically significant.

## 3 Results

### 3.1 Patient Characteristics

**FIGURE 1:**
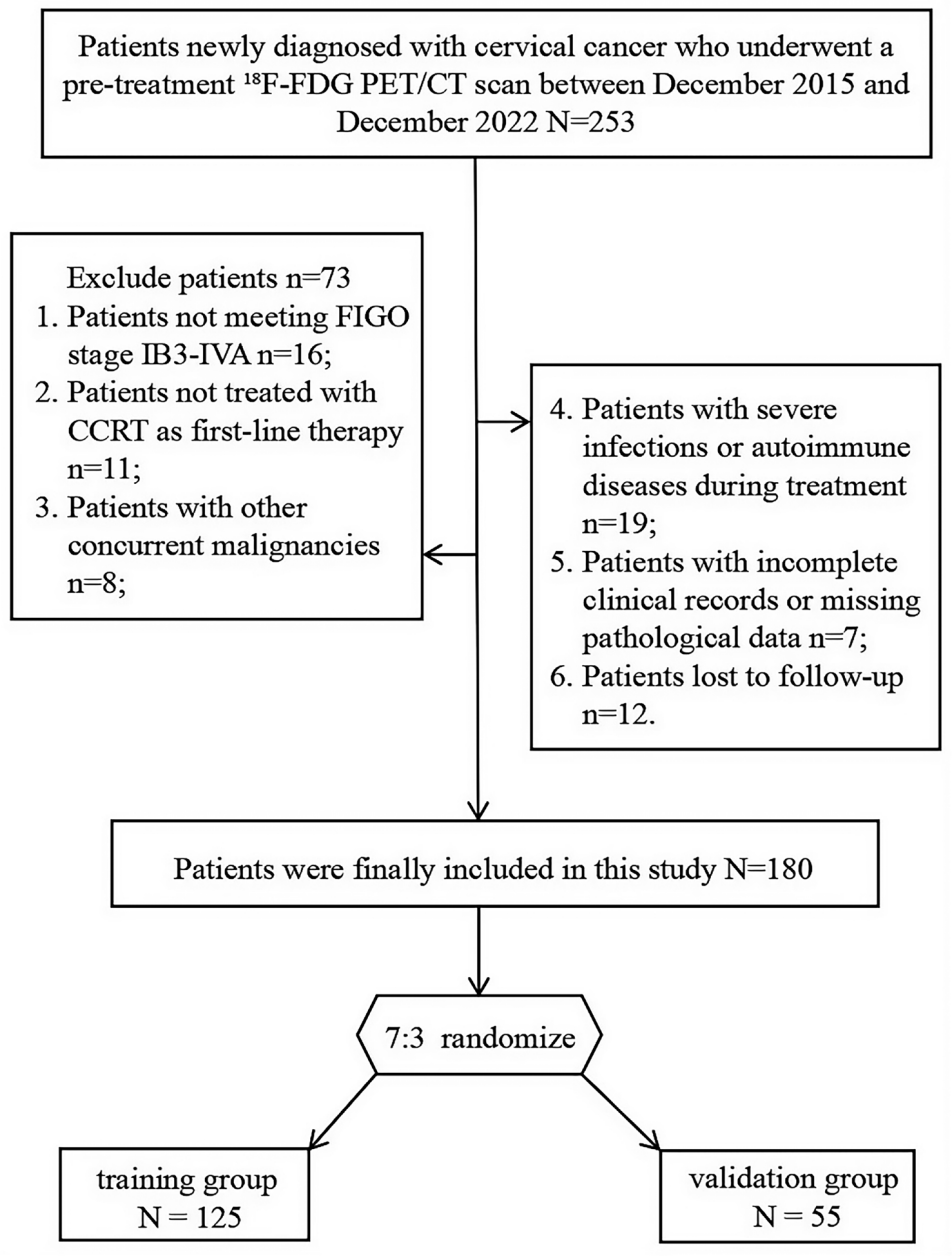
Recruitment and selection process of patients.

A total of 180 patients were included in this study, with randomization into training (*n* = 125) and validation (*n* = 55) groups at a ratio of 7:3 (Figure 1). The baseline characteristics of the groups are summarized in Table 1. There was no statistically significant difference in baseline characteristics between the two groups (Table 1 and Table S1). The median follow‐up time was 52.8 months (range: 4–91.3 months). During this period, 83 patients (46.1%) experienced tumor progression. The 3‐ and 5‐year PFS rates were 61.1% and 56.1%, respectively, while the 3‐ and 5‐year OS rates were 79.4% and 71.1%, respectively.

**TABLE 1:** Patient characteristics.

Variable	All patients	Training cohort (*N* = 125)	Validation cohort (*N* = 55)	*p*
Age, years; mean (range)	53.73 (22–75)	53.51 (22–75)	54.22 (35–75)	0.661
FIGO stage, *n* (%)				0.200
IB3	10 (5.56)	9 (7.20)	1 (1.82)	
IIA‐IIB	55 (30.56)	36 (28.80)	19 (34.55)	
IIIA‐IIIC	103 (57.22)	74 (59.20)	29 (52.73)	
IVA	12 (6.67)	6 (4.80)	6 (10.91)	
MTD, cm; mean (range)	5.54 (4.1–9.8)	5.52 (4.1–9.8)	5.59 (4.1–8.5)	0.609
Pathology, *n* (%)				0.954
SCC	160 (88.89)	111 (88.80)	49 (89.09)	
ADC	20 (11.11)	14 (11.20)	6 (10.91)	
Pelvic LN, *n* (%)				0.403
N0	87 (48.33)	63 (50.40)	24 (43.64)	
N1	93 (51.67)	62 (49.60)	31 (56.36)	
Para‐aortic LN, *n* (%)				0.106
N0	144 (80.00)	104 (83.20)	40 (72.73)	
N1	36 (20.00)	21 (16.80)	15 (27.27)	
EBRT dose, Gy, *n* (%)				0.794
< 50.4	89 (49.44)	61 (48.80)	28 (50.91)	
≥ 50.4	91 (50.56)	64 (51.20)	27 (49.09)	
NLR, mean (range)	3.55 (1.02–11.28)	3.65 (1.34–11.28)	3.33 (1.02–9.79)	0.124
PLR, mean (range)	186.45 (45.38–490.48)	180.54 (45.38–490.48)	199.89 (4.61–386.72)	0.076
LMR, mean (range)	4.06 (1.45–8.95)	4.02 (1.45–8.95)	4.15 (1.49–8.32)	0.635
SII, mean (range)	932.53 (213.87–3776.67)	935.85 (213.87–3776.67)	925.00 (276.81–2849.5)	0.798
SIRI, mean (range)	1.48 (0.31–5.82)	1.52 (0.31–4.96)	1.38 (0.31–5.82)	0.146
MTV, mL, mean (range)	48.58 (2.49–165.18)	49.49 (2.49–165.18)	46.51 (2.62–143.28)	0.960
TLG, mean (range)	412.99 (4.27–2586.31)	406.33 (10.69–2586.31)	428.13 (4.3–2065.34)	0.767
SUV_cervix_, mean (range)	15.99 (1.82–63.2)	15.93 (3.86–36.37)	16.11 (1.82–63.2)	0.207
SUV_spleen_, mean (range)	3.55 (1.24–7.57)	3.45 (2.2–6.35)	3.78 (1.24–7.57)	0.116
SUV_BM_, mean (range)	1.68 (0.44–3.46)	1.66 (0.65–3.02)	1.74 (0.44–3.46)	0.406
SLR, mean (range)	0.97 (0.35–2.12)	0.94 (0.67–1.55)	1.02 (0.35–2.12)	0.296
BLR, mean (range)	0.46 (0.13–1.05)	0.46 (0.19–0.90)	0.47 (0.13–1.05)	0.727

Abbreviations: ADC, adenocarcinoma; BLR, bone marrow‐to‐liver ratio; BM, bone marrow; EBRT, external beam radiation therapy; FIGO, Fédération Internationale de Gynécologie et d'Obstétrique; LMR, Lymphocyte‐to‐Monocyte ratio; MTD, maximum tumor diameter; MTV, metabolic tumor volume; NLR, Neutrophil‐to‐lymphocyte ratio; PLR, Platelet‐to‐lymphocyte ratio; SCC, squamous cell carcinoma; SII, Systemic Immune‐Inflammation Index; SIRI, Systemic Inflammatory Response Index; SLR, spleen‐to‐liver ratio; SUV, standardized uptake value; TLG, total lesion glycolysis.

### 3.2 Prognostic Factor Analysis

Univariable Cox analysis in the training group revealed that FIGO stage, maximum tumor diameter, NLR, SII, SIRI, MTV, TLG, SUV_cervix_, SUV_spleen_, and SLR were significant factors affecting PFS (*p* < 0.05). For OS, significant factors included FIGO stage, maximum tumor diameter, adenocarcinoma, NLR, SII, SIRI, MTV, TLG, SUV_cervix_, SUV_spleen_, and SLR (*p* < 0.05). After multivariable Cox regression analysis, FIGO stage>II, a higher NLR (≥ 3.238), and higher SUV_spleen_ (≥ 3.21) were associated with poorer PFS, while FIGO stage>II, a higher NLR (≥ 3.238), higher MTV (≥ 49.6), and higher SUV_spleen_ (≥ 3.21) were associated with poorer OS (Table 2).

**TABLE 2:** PFS and OS‐related univariable and multivariable analysis.

Variable	PFS				OS			
	Univariable analysis		Multivariable analysis		Univariable analysis		Multivariable analysis	
	HR (95% CI)	*p*	HR (95% CI)	*p*	HR (95% CI)	*p*	HR (95% CI)	*p*
Age (≥ 53 vs. < 53 years)	1.50 (0.87~2.56)	0.141			1.65 (0.84~3.25)	0.144		
FIGO stage (> II vs. ≤ II)	6.84 (2.93~16.00)	**< 0.001**	4.23 (1.72~10.41)	**0.002**	12.36 (2.97~51.43)	**< 0.001**	6.03 (1.29~28.16)	**0.022**
MTD (≥ 5.15 vs. < 5.15 cm)	1.96 (1.10~3.49)	**0.023**	1.18 (0.59~2.37)	0.644	3.32 (1.46~7.56)	**0.004**	2.04 (0.84~4.98)	0.116
Pathology (ADC vs. SCC)	2.01 (0.98~4.10)	0.056			3.04 (1.38~6.70)	**0.006**	1.04 (0.44~2.47)	0.932
Pelvic LN (N1 vs. N0)	1.49 (0.88~2.53)	0.137			1.53 (0.80~2.93)	0.200		
Para‐aortic LN (N1 vs. N0)	1.65 (0.87~3.12)	0.126			1.42 (0.62~3.24)	0.403		
EBRT dose (≥ 50.4 vs. < 50.4 Gy)	0.84 (0.50~1.42)	0.508			0.88 (0.46~1.68)	0.701		
NLR (≥ 3.238 vs. < 3.238)	6.72 (3.35~13.46)	**< 0.001**	4.61 (1.61~13.21)	**0.004**	10.09 (3.55~28.65)	**< 0.001**	6.16 (1.26~30.25)	**0.025**
PLR (≥ 149.54 vs. < 149.54)	1.64 (0.94~2.85)	0.080			1.82 (0.90~3.68)	0.097		
LMR (≥ 6.12 vs. < 6.12)	0.64 (0.27~1.49)	0.303			0.91 (0.35~2.33)	0.843		
SII (≥ 778.5 vs. < 778.5)	3.62 (2.03~6.44)	**< 0.001**	0.51 (0.22~1.18)	0.116	6.12 (2.67~14.00)	**< 0.001**	0.53 (0.16~1.75)	0.295
SIRI (≥ 1.66 vs. < 1.66)	2.99 (1.75~5.09)	**< 0.001**	1.71 (0.92~3.16)	0.088	2.56 (1.34~4.90)	**0.005**	1.14 (0.55~2.33)	0.725
MTV (≥ 49.6 vs. < 49.6 mL)	2.76 (1.63~4.68)	**< 0.001**	1.44 (0.68~3.06)	0.347	6.70 (3.15~14.23)	**< 0.001**	4.18 (1.23~14.23)	**0.022**
TLG (≥ 251.44 vs. < 251.44)	2.43 (1.40~4.24)	**0.002**	0.80 (0.35~1.83)	0.605	4.15 (1.89~9.09)	**< 0.001**	0.35 (0.09~1.37)	0.133
SUV_cervix_ (≥ 11.97 vs. < 11.97)	2.29 (1.08~4.85)	**0.030**	1.09 (0.46~2.59)	0.839	3.35 (1.18~9.48)	**0.023**	2.83 (0.86~9.36)	0.088
SUV_spleen_ (≥ 3.21 vs. < 3.21)	6.63 (2.99~14.67)	**< 0.001**	4.03 (1.66~9.82)	**0.002**	14.10 (3.39~58.66)	**< 0.001**	8.99 (1.76~46.06)	**0.008**
SUV_BM_ (≥ 1.53 vs. < 1.53)	1.56 (0.91~2.68)	0.108			1.93 (0.97~3.84)	0.062		
SLR (≥ 0.935 vs. < 0.935)	5.00 (2.74~9.12)	**< 0.001**	1.89 (0.93~3.87)	0.080	7.37 (3.07~17.73)	**< 0.001**	1.71 (0.56~5.16)	0.345
BLR (≥ 0.64 vs. < 0.64)	1.61 (0.85~3.05)	0.144			1.53 (0.70~3.35)	0.289		

*Note:* Parameters demonstrating statistical significance (*p* < 0.05) are highlighted in bold font.

Abbreviations: ADC, adenocarcinoma; BLR, bone marrow‐to‐liver ratio; BM, bone marrow; EBRT, external beam radiation therapy; FIGO, Fédération Internationale de Gynécologie et d'Obstétrique; LMR, Lymphocyte‐to‐Monocyte ratio; MTD, maximum tumor diameter; MTV, metabolic tumor volume; NLR, Neutrophil‐to‐lymphocyte ratio; PLR, Platelet‐to‐lymphocyte ratio; SCC, squamous cell carcinoma; SII, Systemic Immune‐Inflammation Index; SIRI, Systemic Inflammatory Response Index; SLR, spleen‐to‐liver ratio; SUV, standardized uptake value; TLG, total lesion glycolysis.

Based on these results, we developed predictive models for PFS and OS, which were visualized using nomograms. Additionally, we constructed a FIGO stage model that assesses prognosis based solely on FIGO stage.

### 3.3 Construction and Validation of the Nomogram

**FIGURE 2:**
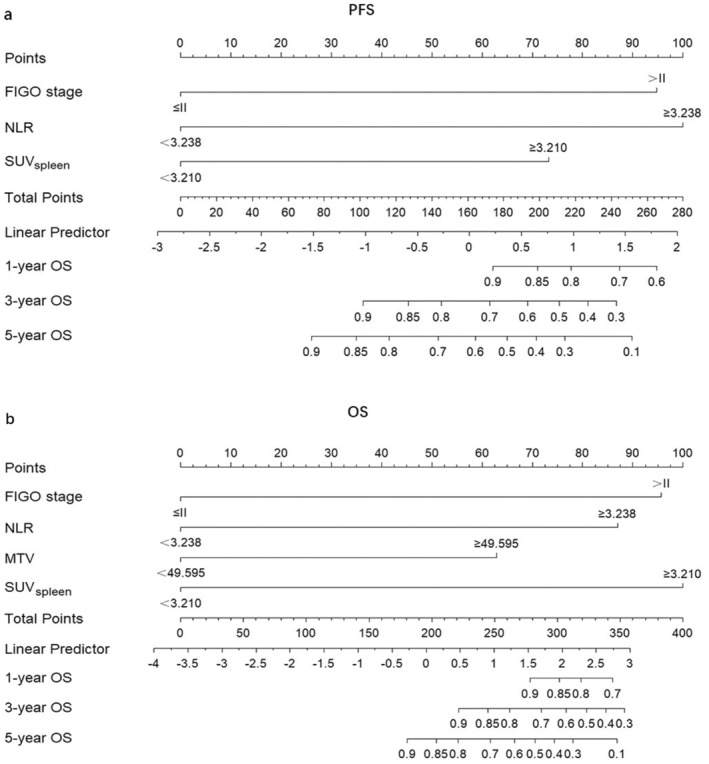
The nomogram for predicting PFS (a) and OS (b) based on clinical parameters and metabolic parameters.

**FIGURE 3:**
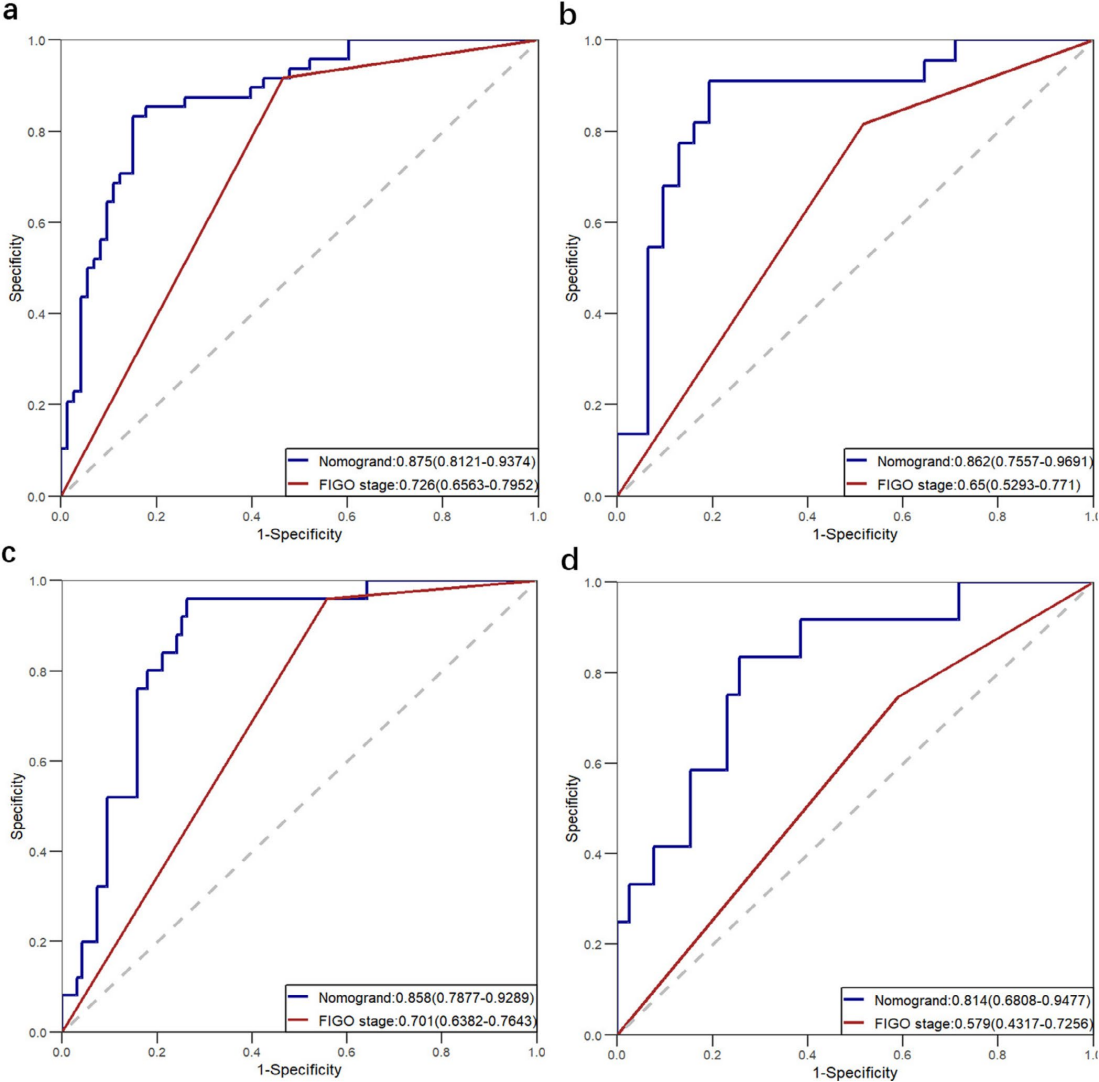
Receiver Operating Characteristic (ROC) curves were used to compare the performance of the nomogram to the FIGO stage. (a) and (b) are the ROC curves for predicting PFS in the training and validation groups, respectively. (c) and (d) are the ROC curves for predicting OS in the training and validation groups, respectively.

**FIGURE 4:**
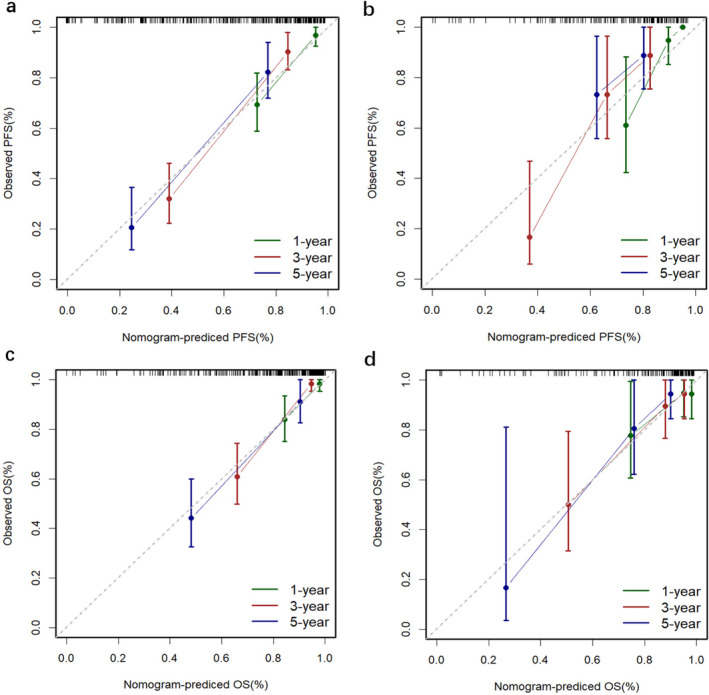
(a) and (b) show the calibration curve of the nomogram for predicting 1‐, 3‐ and 5‐year PFS in the training and validation groups, respectively. (c) and (d) show the calibration curve of the nomogram for predicting 1‐, 3‐ and 5‐year OS in the training and validation groups, respectively.

**FIGURE 5:**
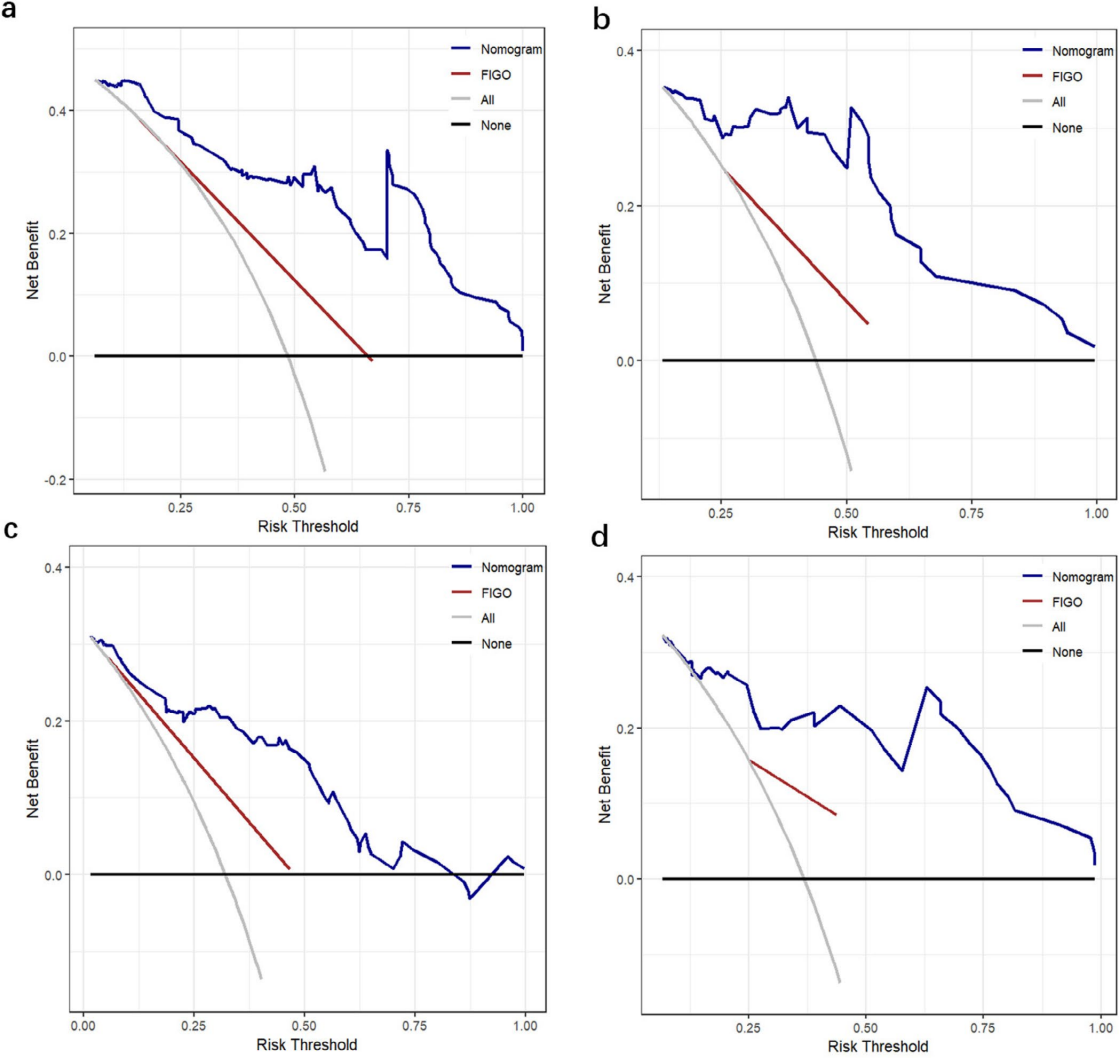
(a) and (b) show the decision curve analysis (DCA) of the nomogram predicting PFS in the training and validation groups, respectively. (c) and (d) show the DCA of the nomogram for predicting OS in the training and validation groups, respectively.

The nomograms for predicting 1‐, 3‐, and 5‐year PFS and OS were developed based on the results of the multivariable Cox regression analysis using the ‘rms’ package in R software (Figure 2). The nomograms demonstrated better predictive performance for predicting PFS (AUC: 0.875 and 0.862; C‐index: 0.809 and 0.775) and OS (AUC: 0.858 and 0.814; C‐index: 0.828 and 0.792) in both the training and validation groups, compared to the FIGO stage model for predicting PFS (AUC: 0.726 and 0.650; C‐index: 0.672 and 0.626) and OS (AUC: 0.701 and 0.579; C‐index: 0.675 and 0.596) in both groups (Figure 3 and Table 3). Calibration curves of the nomograms demonstrated good agreement between predicted and actual outcomes in both the training and validation groups (Figure 4). DCA indicated that the nomograms provided better clinical utility than the FIGO stage model (Figure 5).

**TABLE 3:** The C‐index (95% CI) of the nomogram and FIGO stage model for predicting PFS and OS.

Model	Traininggroup (95% CI)	Validation group (95% CI)
PFS		
FIGO stage	0.672 (0.619~0.725)	0.626 (0.540~0.712)
Nomogram	0.809 (0.758~0.860)	0.775 (0.696~0.854)
OS		
FIGO stage	0.675 (0.619~0.731)	0.596 (0.490~0.702)
Nomogram	0.828 (0.770~0.886)	0.792 (0.679~0.905)

**FIGURE 6:**
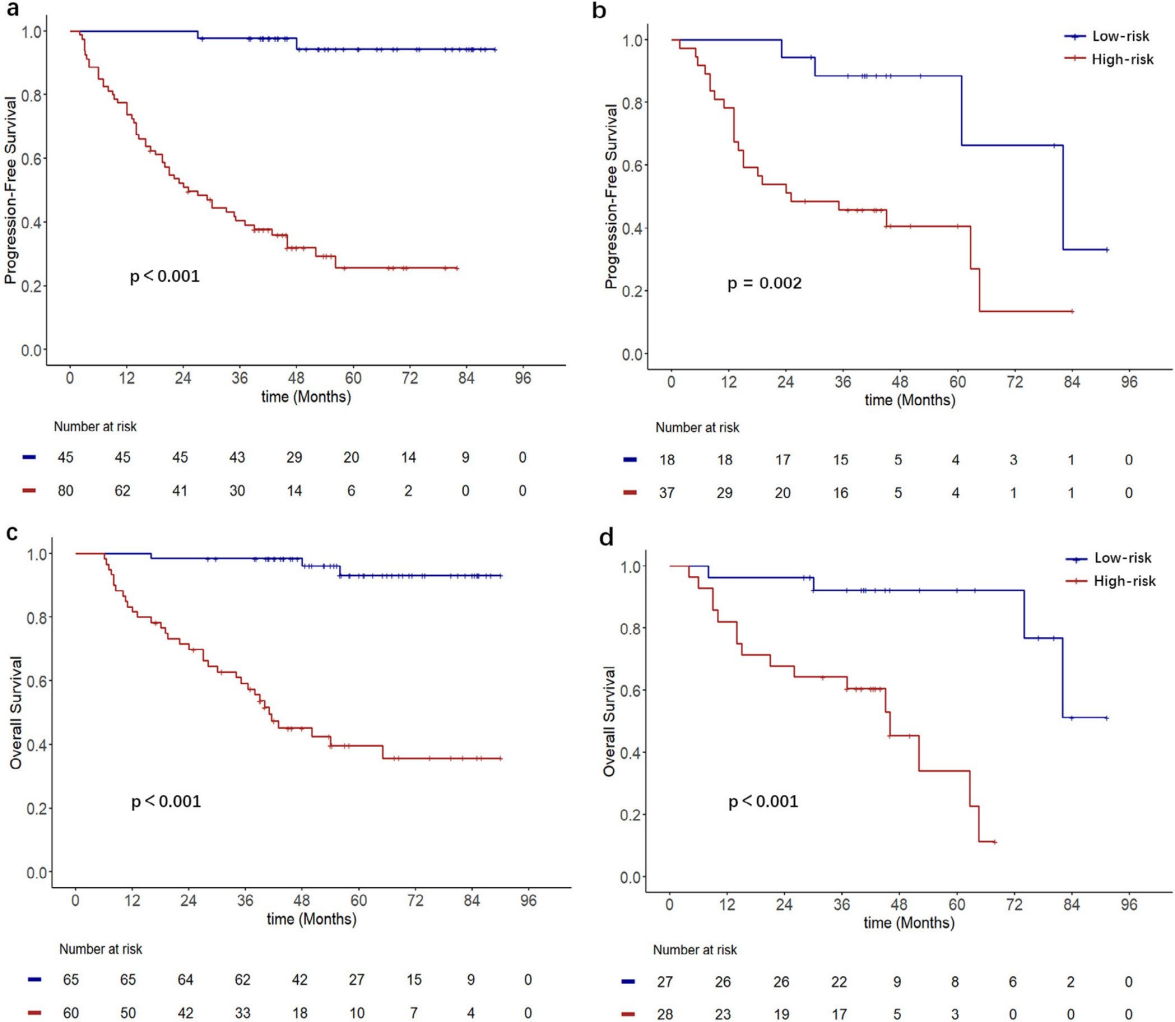
(a) and (b) are the Kaplan–Meier curves for different risk stratifications for PFS in the training group and validation group, respectively. (c) and (d) are the Kaplan–Meier curves for different risk stratifications for OS in the training group and validation group in the training group and validation group, respectively.

The optimal cutoff values of the nomogram scores determined from the ROC curves were 137.6 (nomogram scores based on PFS) and 166.3 (nomogram scores based on OS). Patients were subsequently classified into low‐risk (score < 137.6) and high‐risk (score ≥ 137.6) groups for PFS, as well as low‐risk (score < 166.3) and high‐risk (score ≥ 166.3) groups for OS. Kaplan–Meier analysis indicated that, regardless of PFS or OS, patients in the high‐risk group had worse PFS and OS (Figure 6).

### 3.4 Correlation Analysis

After constructing the nomogram, we explored the correlations between metabolic parameters of different systemic immune organs and their relationships with hematological immune‐related markers using Spearman correlation analysis. The results revealed significant positive correlations among TLG, SUV_cervical_, SUV_spleen_, and SUV_BM_ (*p* < 0.01), particularly between SUV_spleen_ and SUV_BM_ (*R* = 0.49, *p* < 0.001). Following adjustment for SUV_spleen_ and SUV_BM_ using SUV_liver_, the correlation between SLR and BLR was more pronounced (*R* = 0.67, *p* < 0.001).

**FIGURE 7:**
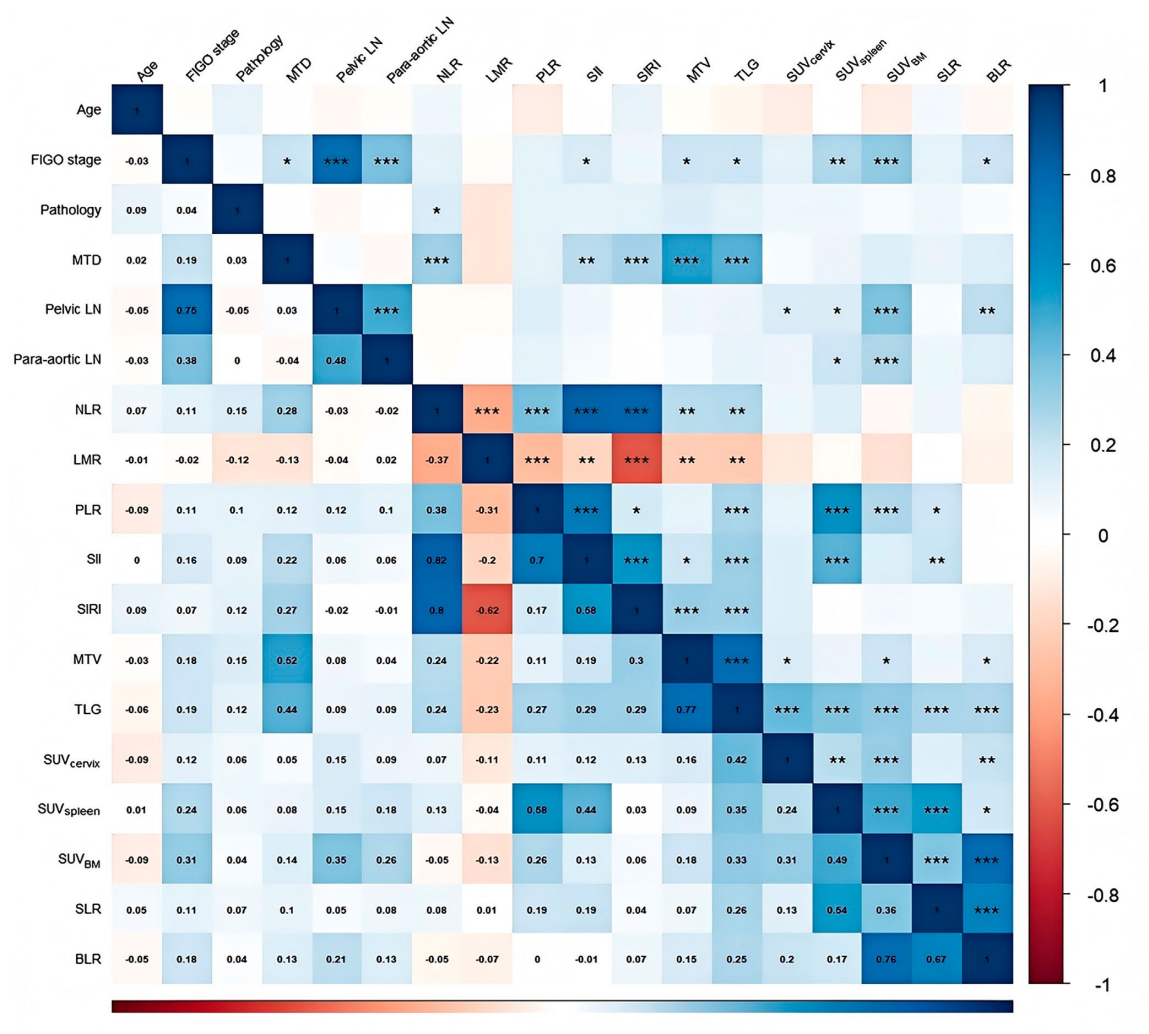
The heatmap showing the associations between all parameters, including clinical and metabolic parameters.

Additionally, hematological immune‐related markers such as NLR, PLR, and SII were significantly positively correlated with TLG (*p* < 0.01), particularly PLR (*R* = 0.58, *p* < 0.001) and SII (*R* = 0.44, *p* < 0.001), which also showed a significant positive correlation with SUV_spleen_ (Figure 7).

## 4 Discussion

We developed nomograms that integrate hematological immune‐related markers and immune‐related metabolic parameters to estimate the probabilities of PFS and OS in patients with LACC who underwent CCRT. Our findings demonstrated that the nomograms have superior predictive performance compared to the FIGO stage model. Additionally, we observed correlations among the hematological markers and the metabolic parameters of the primary cervical tumor, spleen, and bone marrow, emphasizing the interconnections among these immune organs and systemic immunity. To the best of our knowledge, this is the first study to comprehensively evaluate the hematological immune‐related markers and metabolic parameters of both the primary tumor and immune organs in patients with LACC who underwent CCRT. The prognostic stratification model developed based on these factors not only facilitates a quantitative analysis of the tumor's biological characteristics but also provides a comprehensive reflection of the patient's systemic immune status.

With the widespread application of ^18^F‐FDG PET/CT, the prognostic significance of metabolic parameters has gained increasing attention. Currently, parameters such as the SUV_max_ of the primary tumor, MTV, and TLG have been established as prognostic indicators in cervical cancer [18, 34]. Additionally, the metabolism of the spleen and bone marrow has been shown to be associated with both prognosis and systemic immunity [15, 16]. Similar findings have been reported in other types of cancer. Schwenck et al. [35] observed a significant increase in glucose metabolism in the bone marrow of patients with melanoma who had a clinical response to checkpoint inhibitor therapy. Seith et al. [36] found increased spleen uptake among responders and identified a correlation between spleen SUV_mean_ and baseline immune biomarkers, such as the NLR and relative lymphocyte count, in patients with melanoma undergoing checkpoint inhibitor therapy. Similarly, we found significant correlations between TLG and NLR, PLR, and SII. Notably, the correlation between SUV_spleen_ and PLR is particularly strong. These findings highlight that, to some extent, ^18^F‐FDG PET/CT may effectively monitor systemic immunity in patients.

Systemic immunity plays a crucial role in cancer prevention and control. The tumor dynamically reshapes the composition and function of systemic immunity throughout its progression, leading to an immunosuppressive state that increases the risk of recurrence and metastasis [11, 19]. Additionally, it is noteworthy that disturbances in systemic immunity caused by tumors often involve changes across multiple organs. Allen et al. [20] observed that during tumor growth, the total count of tumor‐infiltrating leukocytes was positively correlated with both tumor size and increased spleen cell count. Additionally, dynamic changes were noted in the immune cell states of various immune organs, while the immune composition in the spleen, bone marrow, and blood remained relatively consistent. We analyzed the correlations in metabolism between these organs and found significant associations between the primary cervical tumor, spleen, and bone marrow. Notably, we observed a more significant positive correlation between SUV_spleen_ and SUV_BM_. After adjusting for these parameters using SUV_liver_, we calculated the SLR and BLR and found an even more pronounced correlation between SLR and BLR. This suggested that SLR and BLR might be more stable metabolic parameters for assessing and comparing systemic immunity across different patients.

During tumor progression, patients often experience anemia and abnormal hematopoiesis in the bone marrow, which can increase bone marrow metabolism [37, 38]. The spleen, as a primary site of extramedullary hematopoiesis (EMH), plays a compensatory role in diseases such as malignancies and anemia, which may lead to increased metabolic activity [39]. This functional compensation of the bone marrow by the spleen could enhance the metabolic correlation between these two organs. Additionally, the increased metabolic activity of the spleen caused by EMH is also one of the reasons for poor prognosis in patients. During this process, a significant proportion of erythroid progenitor cells (EPCs) differentiates into erythroid differentiated myeloid cells under the influence of tumors, leading to immune suppression and promoting tumor progression [40]. As our results indicated, a higher SUV_spleen_ was associated with an increased risk of recurrence and poorer OS in patients with LACC.

Systemic inflammatory responses can also elevate the metabolism of the immune‐related organs, including the spleen and bone marrow [15, 41, 42, 43]. Our results show that hematological markers, such as PLR and SII, were associated with the metabolism of these organs, particularly with a significant positive correlation to SUV_spleen_. The spleen is crucial for recruiting and activating inflammation‐related immune cells and driving pro‐inflammatory immune responses [44, 45]. Increased glycolytic activity in tumor‐promoting immune cells, such as regulatory T cells or tumor‐associated macrophages, may be a primary reason for the increased ^18^F‐FDG uptake in the spleen during inflammatory responses [15]. Lin et al. [46] evaluated immune activity in patients with cervical cancer using HP [1‐^13^C]‐pyruvate magnetic resonance spectroscopy (MRS) of the spleen. They discovered that the baseline splenic HP [1‐^13^C]‐lactate‐to‐total carbon (tC) ratio was significantly lower in responders compared to non‐responders. Furthermore, they proposed that the increase in the splenic lactate‐to‐tC ratio after radiotherapy was primarily associated with potential immune activation. This emphasizes the potential of non‐invasive metabolic monitoring of immune organs such as the spleen and bone marrow to provide new methods for assessing systemic immunity and predicting patient prognosis, providing clinicians with more comprehensive insights for decision‐making.

The clinical significance of this study is that we proposed a multidimensional approach to prognosis assessment, incorporating tumor burden (staging and metabolism) and systemic immune status. Specifically, nomograms based on clinical characteristics, hematological immune‐related markers, and metabolic parameters of both the primary tumor and immune organs significantly enhance the accuracy of prognosis predictions and offer greater clinical utility compared to traditional FIGO staging models. Increased metabolism in the spleen reflects a higher risk of disease recurrence in patients with LACC, suggesting that an intensive surveillance strategy focused on the spleen could improve clinical outcomes. Additionally, the metabolic correlations among different immune organs indicate that clinicians should make decisions based on a comprehensive assessment of systemic immunity.

Our study has several limitations. First, it is a single‐center retrospective study with a limited number of patients; thus, selection bias is inevitable. Larger‐scale external validation is needed to confirm our findings. Future research will involve collaboration with multiple centers to validate the practical utility of our nomograms. Second, although we did not find significant prognostic value in bone marrow metabolism, we identified a relationship between bone marrow metabolism and hematological markers. Further validation is required through bone marrow aspiration and serum cytokine levels. Finally, these immune‐related prognostic parameters and models may only apply to patients receiving CCRT, as different treatment modalities may have varying impacts on the tumor microenvironment, systemic immunity, and survival outcomes. This aspect requires further validation.

## 5 Conclusions

The nomograms based on hematological immune‐related markers and immune‐related metabolic parameters demonstrate high predictive value for patients with LACC. The correlations observed between the metabolic parameters of different immune organs emphasize the widespread disruption of systemic immunity in tumor‐bearing patients. Interpreted through this model, these findings may serve as predictors of cancer progression and guiding treatment decisions.

## Author Contributions

**Yi Li:** conceptualization (equal); data curation (equal); writing – original draft (equal). **Ao Liu:** conceptualization (equal); writing – original draft (equal). **Xin Wang:** conceptualization (equal); visualization (equal); writing – original draft (equal). **Yuanlin Li:** data curation (equal). **Zhichao Li:** visualization (equal); writing – original draft (equal). **Xiuli Liu:** data curation (equal); formal analysis (equal). **Wanhu Li:** data curation (equal); formal analysis (equal); software (equal); visualization (equal). **Defeng Liu:** writing – original draft (equal). **Longxiang Guo:** conceptualization (equal); writing – review and editing (equal). **Minghuan Li:** conceptualization (equal); writing – review and editing (equal).

## Ethics Statement

Our retrospective study abided by the rules of medical ethics, and the Institutional Review Board (IRB) of Shandong Cancer Hospital approved this study. The number for the ethical statement was SDTHEC2024006159. All patients were informed before treatment, agreed to receive concurrent CCRT and signed informed consent forms. We protected patient privacy and excluded patient identification information from our analyses.

## Conflicts of Interest

The authors declare no conflicts of interest.

## Supporting information


Table S1.

## Data Availability

The data underlying this article cannot be shared publicly due to this study is based on registry data from Shandong Cancer Hospital and Research Institute, which the authors do not own. The data will be shared on reasonable request to the corresponding author.
