# Supplementary material for: The Combined Prognostic Value of 18 F‐FDG PET/CT Metabolic Parameters of Immune Organs and Hematological Immune‐Related Markers in Patients With Locally Advanced Cervical Cancer
Source: Cancer Med. 2025 Feb 6;14(3):e70650. doi: 10.1002/cam4.70650 (PMC11800136; doi:10.1002/cam4.70650)
Supplement: Supplementary file 1 — Table S1. [file CAM4-14-e70650-s001.docx]

| Variable | Cutoff | Total | Training cohort | Validation cohort | P-value |
| --- | --- | --- | --- | --- | --- |
|  |  | (N = 180) | (N = 125) | (N = 55) |  |
| Age, years, n (%) | < 53 | 81 (45.00) | 58 (46.40) | 23 (41.82) | 0.569 |
|  | ≥ 53 | 99 (55.00) | 67 (53.60) | 32 (58.18) |  |
| FIGO stage, n (%) | ≤ II | 65 (36.11) | 45 (36.00) | 20 (36.36) | 0.963 |
|  | > II | 115 (63.89) | 80 (64.00) | 35 (63.64) |  |
| MTD, cm, n (%) | < 5.15 | 72 (40.00) | 52 (41.60) | 20 (36.36) | 0.509 |
|  | ≥ 5.15 | 108 (60.00) | 73 (58.40) | 35 (63.64) |  |
| NLR, n (%) | < 3.238 | 87 (48.33) | 56 (44.80) | 31 (56.36) | 0.153 |
|  | ≥ 3.238 | 93 (51.67) | 69 (55.20) | 24 (43.64) |  |
| PLR, n (%) | < 149.54 | 67 (37.22) | 52 (41.60) | 15 (27.27) | 0.067 |
|  | ≥ 149.54 | 113 (62.78) | 73 (58.40) | 40 (72.73) |  |
| LMR, n (%) | < 6.12 | 155 (86.11) | 108 (86.40) | 47 (85.45) | 0.866 |
|  | ≥ 6.12 | 25 (13.89) | 17 (13.60) | 8 (14.55) |  |
| SII, n (%) | < 778.5 | 89 (49.44) | 64 (51.20) | 25 (45.45) | 0.478 |
|  | ≥ 778.5 | 91 (50.56) | 61 (48.80) | 30 (54.55) |  |
| SIRI, n (%) | < 1.66 | 119 (66.11) | 80 (64.00) | 39 (70.91) | 0.367 |
|  | ≥ 1.66 | 61 (33.89) | 45 (36.00) | 16 (29.09) |  |
| MTV, mL, n (%) | < 49.6 | 106 (58.89) | 76 (60.80) | 30 (54.55) | 0.432 |
|  | ≥ 49.6 | 74 (41.11) | 49 (39.20) | 25 (45.45) |  |
| TLG, n (%) | < 251.44 | 88 (48.89) | 60 (48.00) | 28 (50.91) | 0.719 |
|  | ≥ 251.44 | 92 (51.11) | 65 (52.00) | 27 (49.09) |  |
| SUV_cervix_, n (%) | < 11.97 | 48 (26.67) | 30 (24.00) | 18 (32.73) | 0.223 |
|  | ≥ 11.97 | 132 (73.33) | 95 (76.00) | 37 (67.27) |  |
| SUV_spleen_, n (%) | < 3.21 | 61 (33.89) | 48 (38.40) | 13 (23.64) | 0.054 |
|  | ≥ 3.21 | 119 (66.11) | 77 (61.60) | 42 (76.36) |  |
| SUV_BM_, n (%) | < 1.53 | 78 (43.33) | 57 (45.60) | 21 (38.18) | 0.355 |
|  | ≥ 1.53 | 102 (56.67) | 68 (54.40) | 34 (61.82) |  |
| SLR, n (%) | < 0.935 | 88 (48.89) | 65 (52.00) | 23 (41.82) | 0.208 |
|  | ≥ 0.935 | 92 (51.11) | 60 (48.00) | 32 (58.18) |  |
| BLR, n (%) | < 0.64 | 150 (83.33) | 105 (84.00) | 45 (81.82) | 0.717 |
|  | ≥ 0.64 | 30 (16.67) | 20 (16.00) | 10 (18.18) |  |

**Supplementary Table 1** Patient characteristics (categorical variables)

Abbreviations: FIGO, Fédération Internationale de Gynécologie et d'Obstétrique; MTD, maximum tumor diameter; SCC, squamous cell carcinoma; ADC, adenocarcinoma; EBRT, external beam radiation therapy; NLR, Neutrophil-to-lymphocyte ratio; PLR, Platelet-to-lymphocyte ratio; LMR, Lymphocyte-to-Monocyte ratio; SII, Systemic Immune-Inflammation Index; SIRI, Systemic Inflammatory Response Index; MTV, metabolic tumor volume; TLG, total lesion glycolysis; SUV, standardized uptake value; BM, bone marrow; SLR, spleen-to-liver ratio; BLR, bone marrow -to-liver ratio
